# Clinical and Radiological Insights of Cleidocranial Dysplasia: A Case Report of a Rare Medical Condition

**DOI:** 10.7759/cureus.64456

**Published:** 2024-07-13

**Authors:** Ramachandra Reddy Gowda Venkatesha, Karthik Rajaram Mohan, Mirnalini Aravind, Vasu Sridharrao, Sindhuja Rajalingam

**Affiliations:** 1 Department of Oral Medicine and Radiology, Vinayaka Mission's Sankarachariyar Dental College, Vinayaka Mission's Research Foundation (Deemed to be University), Salem, IND

**Keywords:** pubic symphysis diastasis, coxa valga, cone-beam computed tomography (cbct), panoramic radiograph, dental anomalies, cleidocranial dysplasia (ccd)

## Abstract

Cleidocranial dysplasia (CCD) is a rare, congenital disorder characterized by a unique constellation of skeletal and dental abnormalities. The imaging findings, combined with clinical examination, help establish a definitive diagnosis. Understanding the broad spectrum of manifestations in CCD is essential for effective management and treatment. This case report aims to provide a comprehensive overview of a 25-year-old male patient with CCD, highlighting the genetic etiologies, clinical presentation, radiological findings, and a review of current literature to enhance understanding and awareness of this rare condition.

## Introduction

Cleidocranial dysplasia (CCD) is a skeletal condition inherited in an autosomal dominant manner [1]. In 1898, Marie and Sainton were the first to report on CCD [1]. Both men and women are equally affected by CCD, which has a prevalence rate of one per one million individuals and has a tendency to be passed down from generation to generation [1]. CCD is caused by a mutation in the RUNX2 gene, characterized by skeletal abnormalities that affect both the bones and the teeth [1]. Additional activities depend on the gene, including condensation and osteoblast differentiation from mesenchymal stem cells (MSCs), chondrocyte hypertrophy, and vascular invasion in the growing skeleton [1,2]. The gene that causes the condition is located on chromosome 6p21, specifically in a region that contains CBFA1, which is a transcription factor belonging to the Runt protein family [2]. One of the most significant dental concerns for these patients is the incomplete or delayed eruption of permanent teeth, which is one of the many indications of this orthodontic condition. To provide complete and patient-centered care, dental physicians, orthodontists, endodontists, and prosthodontists must thoroughly understand this genetic illness's complexities. An approach incorporating multiple disciplines is required to manage CCD patients [1,2]. The goal of this case report is to detail the clinical and radiological features of CCD.

## Case presentation

**Figure 1:**
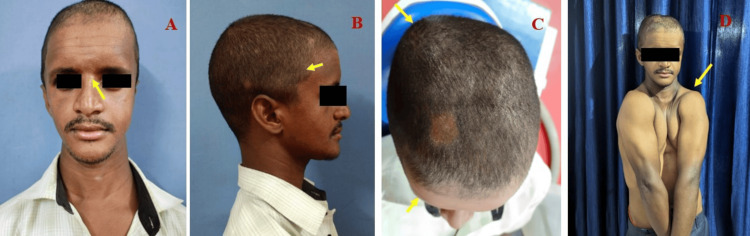
(A) Extraoral examination revealing increased intercanthal distance (hypertelorism) (yellow arrow). (B) Frontal bossing (yellow arrow). (C) Frontal and parietal bossing (yellow arrow). (D) Ability to approximate shoulder girdle due to the absence of clavicle (yellow arrow)

A 25-year-old male reported to our Department of Oral Medicine and Radiology for a routine dental checkup. General examination revealed a short stature height of 144 cm, weight of 45 kg, frontal bossing, and increased intercanthal distance (hypertelorism) (Figures 1A, 1B).

**Figure 2:**
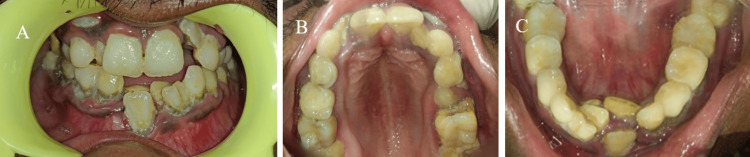
Intraoral clinical examination showing (A) multiple overretained deciduous teeth and multiple missing permanent teeth, (B) high vault palate in the maxilla, and (C) multiple overretained deciduous teeth in the mandible

Intraoral examination revealed multiple missing permanent and retained deciduous teeth (Figures 2A-2C).

**Figure 3:**
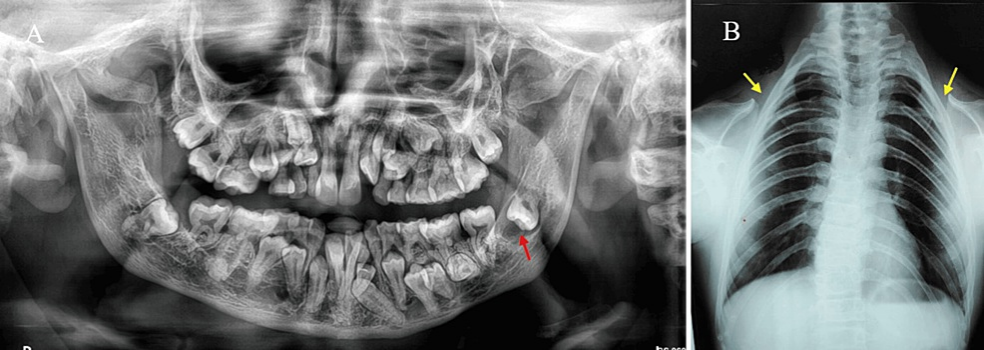
(A) Orthopantomography revealing multiple impacted permanent teeth and a dentigerous cyst around 38 in the ascending ramus of the mandible (red arrow). (B) Chest X-ray revealing the absence of a clavicle (yellow arrow)

The provisional diagnosis includes Gardner's syndrome, Yunis-Varon syndrome, and Gorlin-Sedano syndrome. Gardner's syndrome is characterized by multiple osteomas with impacted supernumerary teeth. Gorlin-Goltz syndrome is characterized by multiple impacted teeth, multiple dentigerous cysts, calcification of falx cerebelli, and multiple basal cell nevus carcinoma. Yunis-Varon syndrome is characterized by clavicular hypoplasia, sparse eyebrows, midfacial hypoplasia, and micrognathia. The differential diagnosis includes Gardner's syndrome, in which multiple impacted supernumerary teeth are associated with numerous odontomas and osteomas. Orthopantomography revealed multiple impacted permanent teeth, retained deciduous teeth, and a circumferential cystic lesion encircling the crown of the impacted left mandibular third molar, which is pushed into the ascending ramus of the mandible, suggestive of dentigerous cyst. The radiographic differential diagnosis includes Gorlin-Goltz syndrome or basal cell naevi-bifid rib syndrome. The chest radiograph revealed an absence of clavicle on both sides and lateral bending (scoliosis) of vertebrae (Figures 3A, 3B).

**Figure 4:**
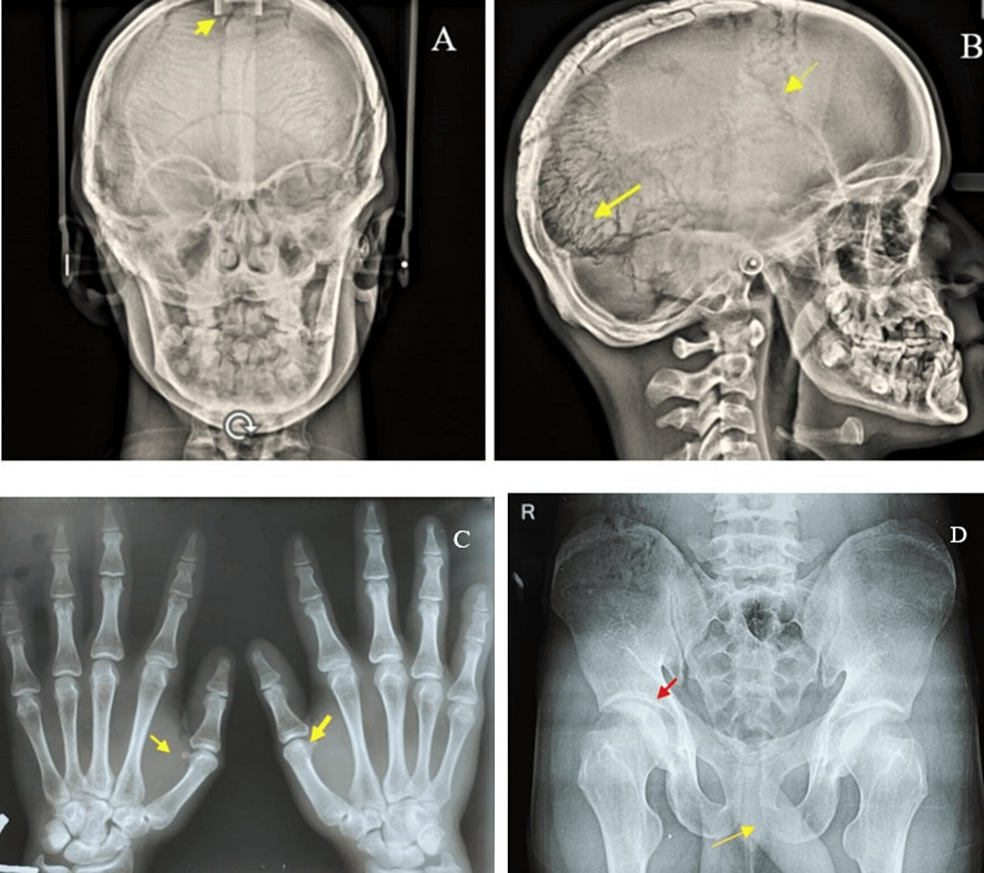
(A) Anteroposterior skull radiograph revealing a lack of closure of sutures on the skull, resulting in open fontanelles (yellow arrow). (B) Lateral skull radiograph revealing increased sutural markings on the skull and nonunion of the cranial bone (yellow arrow). (C) Hand-wrist radiograph revealing metaphyseal deformities of the first metacarpophalangeal bone in both the right and left forearms (yellow arrow). (D) Pelvic X-ray revealing coxa valga (red arrow) and increased pubic symphysis diastasis (yellow arrow)

An anteroposterior skull view revealed nonclosure of cranial sutures, resulting in open fontanelles and increased suture markings, as well as widened cranial sutures due to multiple wormian bones on the skull. A hand-wrist radiograph revealed metaphyseal deformities of the first metacarpophalangeal bone in both the right and left forearms. A pelvic X-ray revealed coxa valga and pubic symphysis diastasis (Figures 4A-4D).

**Figure 5:**
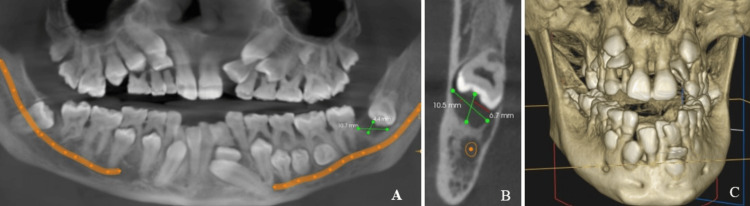
(A) CBCT panoramic mode at a slice thickness of 9.9 mm revealed multiple retained deciduous teeth, multiple impacted permanent teeth, and a 4.4 mm x 10.7 mm circumferential radiolucency encircling the crown of the left mandibular third molar. (B) CBCT coronal section revealed a well-defined circumferential radiolucency measuring about 10.5 mm x 6.7 mm around the crown of the left mandibular third molar. (C) 3D-reconstructed CBCT image also showed multiple retained deciduous teeth and impacted permanent teeth CBCT: cone-beam computed tomography

A panoramic mode cone-beam computed tomography image with a slice thickness of 9.9 mm revealed multiple retained deciduous teeth, impacted permanent teeth, and a well-defined circumferential radiolucency measuring about 10.7 mm x 4.4 mm around the crown of the left mandibular third molar. This molar is pathologically pushed superiorly, which resulted in an inverted direction of the crown, and migrated pathologically along the vertical aspect involving the ascending ramus of the left mandible due to the pressure effects caused by a circumferential dentigerous cyst on the teeth (Figures 5A, 5B).

The patient was advised of dental extraction of retained primary teeth and orthodontic guidance of underlying permanent teeth. Counseling was provided to the patient about the complications that could ensue if the impacted teeth were not removed, and the patient was referred to a multidisciplinary team comprising oral surgeons, prosthodontists, and orthodontists to correct malocclusion and achieve a harmonious occlusion.

## Discussion

Definition

CCD is a rare genetic disorder affecting the skull bone and dentofacial structures [2].

Etymology

The word "cleido" refers to collar bones, "cranial" refers to the skull, "plasia" is derived from modern Latin and the Greek word, "plassein" means to mold or form, and "dysplasia" refers to incomplete form [2].

Etiologies

CCD is the result of a mutation in the Runt-related transcription factor 2 (RUNX2) gene, found explicitly on locus 21 of the short arm (p) of chromosome 6 (6p21). The expression of FBXW11 is regulated during the process of bone formation. It is found to be highly expressed in circulating MSCs as well as in cells that are prompted to undergo osteogenesis in individuals with CCD [3]. Genomic DNA analysis using Sanger sequencing identified three new heterozygous variants (c.538_539delinsCA, c.651_652delTA, and c.662T>A) and an old variant (c.674G>A) in patients with CCD [3].

Clinical features

CCD is a skeletal dysplasia of genetic origin that affects a small proportion of the population, with a prevalence ranging from one to nine cases per 100,000 individuals [3]. The cranial features of CCD include frontal and parietal bossing, increased intercanthal distance (hypertelorism), and short, stubby fingers. A combination of nasal bone hypoplasia, skull and clavicle hypoplasia, and shortened long bones may be an efficient method for early diagnosis of CCD [4]. CCD is a condition marked by reduced bone tissue production caused by genetic abnormalities in the RUNX2 gene C-terminal region [5].

The functional consequences of the cells transfected with different variants located in the C-terminus of RUNX2, including both the proline-serine-threonine-rich (PST) and nuclear matrix targeting sequence regions, were changed. Additionally, the osteogenic capability of primary alveolar bone MSCs harboring the p.Ser247Valfs*3 in PST was also altered. We have shown that the functional consequences of RUNX2 mutations are modified by the precise position and nature of the mutation (truncating or missense), as well as the types of cells employed in the investigation [6]. Peptidylarginine deiminases (PADIs) are enzymes that require calcium to break down peptidyl-arginine into peptidyl-citrulline. This process is called protein citrullination or deamination [7]. The molecular mechanism through which PADI2 controls osteoblast function offers novel insights into the causes of CCD and the potential development of treatments for bone abnormalities [7].

In our case, we also observed a downward curvature of the zygomatic arch in the digital orthopantomography image, as reported by Shi et al. [8]. Supernumerary molars are a rare abnormality that can arise in the maxillofacial complex, indicating the existence of extra teeth in the dental arch. This phenomenon is frequently linked to uncommon illnesses, such as CCD [8]. The use of whole genome sequencing led to the identification of a microdeletion of 11.38 kb in the RUNX2 gene responsible for causing CCD. Deletion of the PST domain in RUNX2 diminishes its transcriptional activity and lowers the levels of osteogenic markers, ultimately leading to a decrease in osteoblast development. These findings elucidate the mechanism of CCD progression [9]. The malfunction of osteoclasts, caused by ovarian cancer long noncoding RNA (OC-lncRNA), is mediated through the OC-lncRNA-miR-221-5p CXCR3 axis. This axis is involved in the late onset of an eruption of the tooth in CCD [10]. Multiple dentigerous cysts occur bilaterally in patients with CCD [11]. Shinde et al. stated that a personalized treatment strategy is necessary to effectively resolve the delicate nature of impaction. The combined efforts of orthodontic and surgical specialists and the step-by-step application of orthodontic and surgical phases have significantly transformed the treatment of impacted supernumerary teeth. This collaborative approach not only leads to a notable enhancement in the visual appeal, usability, and overall oral health but also represents substantial progress in the development of dental treatment [12]. CCDs are associated with double mesiodens [13]. Our case is similar to the orthopantomographic findings of multiple overretained primary teeth and multiple impacted supernumerary teeth, which is an early key finding in the diagnosis of CCD, as stated by Koduru Laxmi et al. [14]. CCD is characterized by multiple impacted permanent teeth [15]. Cissé et al. reported that a novel mutation in the RUNX2 gene resulted in a rare skeletal dysplasia called CCD in a 20-month-old Malibu girl from Africa [16]. The facial features of CCD, in our case, include bossing or bulging of frontal and parietal bones, increased intercanthal distance (hypertelorism), midfacial hypoplasia, and depressed nasal bridge. The orodental features include a high-arched palate, malocclusion, anterior open bite, multiple retained deciduous teeth, multiple impacted permanent teeth, and skeletal class III malocclusion. Extraoral radiological features include the absence of clavicle, scoliosis, wide pubic ectasis, chef hat deformity of the femoral head, widened angle of the sacroiliac joint, and coxa valga of the hip joint (>135°). Intraoral radiological features include multiple retained deciduous teeth and multiple impacted permanent teeth. When combined with polyethylene glycol, lysozyme produces a remineralization effect in the hypoplastic dentin of patients with CCD [17]. Fisetin promotes the maturation of osteoblasts in cell cultures derived from patients with CCD. Notably, poly(lactic-co-glycolic acid) nanoparticles enhanced the stability of fisetin and, as a result, their ability to stimulate the production of RUNX2 and its downstream gene SP7 or Osterix. Their research revealed fisetin's beneficial impacts on bone formation, known as osteogenesis [18]. The periapical scar resulted as a result of the removal of unerupted supernumerary teeth in a CCD patient mimicked as symptomatic apical periodontitis in a root canal-treated teeth, making the endodontist identify it as an endodontic failure mistakenly [19]. The ultrastructural examination demonstrated surface roughness of enamel, measured using a surface profilometer in patients with CCD [20]. Histological analysis using scanning electron microscopy indicated a flat dentino enamel connection. Using energy-dispersive X-ray analysis showed a decrease in the microhardness of enamel and dentin. Additionally, the application of microcomputerized computed tomography (CT) scanning to assess mineral density revealed a lower calcium/phosphate ratio in individuals with CCD [20].

## Conclusions

Dental professionals must thoroughly understand the clinical and radiographic characteristics of the CCD condition. Early diagnosis and extensive care are crucial for addressing the unique needs of individuals with CCD and enhancing their quality of life. This case report highlights the clinical and radiological features of CCD, and dentists must be aware of such findings and guiding a proper treatment.
